# The relationship between ambulatory arterial stiffness, inflammation, blood pressure dipping and cardiovascular outcomes

**DOI:** 10.1186/s12872-021-01946-2

**Published:** 2021-03-16

**Authors:** Christopher J. Boos, Lin-Thiri Toon, Halah Almahdi

**Affiliations:** 1 Department of Cardiology, University Hospital Dorset NHS Foundation Trust, Longfleet Rd., Poole, BH15 2JB UK; 2 Department of Postgraduate Medical Education, Bournemouth University, Bournemouth, BH1 3LT UK; 3 Research Institute, for Sport, Physical Activity and Leisure, Leeds Beckett University, Leeds, LS16 5LF UK

## Abstract

**Background:**

The ambulatory arterial stiffness index (AASI) is an indirect measure of arterial stiffness obtained during ambulatory blood pressuring monitoring (ABPM). Its relationship to nocturnal blood pressure dipping status and major adverse cardiovascular events (MACE) are controversial and its association with vascular inflammation has not been examined. We aimed to investigate the relationship between the AASI, inflammation and nocturnal blood pressure dipping status and its association with MACE.

**Methods:**

Adults (aged 18–80 years) who underwent 24-h ABPM for the diagnosis of hypertension or its control were included. The inflammatory markers measured were the neutrophil–lymphocyte (NLR), platelet-lymphocyte (PLR) and monocyte-lymphocyte ratios (MLR). The primary MACE was a composite of cardiovascular death, acute limb ischaemia, stroke or transient ischaemic attack (TIA) or acute coronary syndrome.

**Results:**

A total of 508 patients (51.2% female) aged 58.8 ± 14.0 years were included; 237 (46.7%) were normal-dippers (≥ 10% nocturnal systolic dip), 214 (42.1%) were non-dippers (0–10% dip) and 57 (11.2%) were reverse-dippers (< 0% dip). The AASI was significantly higher among reverse (0.56 ± 0.16) and non-dippers (0.48 ± 0.17) compared with normal dippers (0.39 ± 0.16; *p* < 0.0001) and correlated with the NLR (r = 0.20; 95% CI 0.11 to 0.29: < 0.0001) and systolic blood pressure dipping % (r = − 0.34; − 0.42 to − 0.26: *p* < 0.0001). Overall 39 (7.7%) patients had ≥ 1 MACE which included a total of seven cardiovascular deaths and 14 non-fatal strokes/TIAs. The mean follow up was 113.7 ± 64.0 weeks. Increasing NLR, but not AASI or systolic dipping, was independently linked to MACE (overall model Chi-square 60.67; *p* < 0.0001) and MLR to cardiovascular death or non-fatal stroke/TIA (overall model Chi-square 37.08; *p* < 0.0001).

**Conclusions:**

In conclusion AASI was associated with blood pressure dipping and chronic inflammation but not independently to MACE. The MLR and NLR were independent predictors of MACE.

**Supplementary Information:**

The online version contains supplementary material available at 10.1186/s12872-021-01946-2.

## Introduction

Twenty four hour ambulatory blood pressure (ABP) monitoring (ABPM) represents the gold standard for the diagnosis of hypertension and assessment of its control [1–3]. Among the routinely reported ABPM variables the ambulatory arterial stiffness index (AASI) and blood pressure dipping status have gained increasing research interest. Non-dipping of nocturnal blood pressure has been independently linked to target organ damage and major adverse cardiovascular events (MACE) including stroke, cardiovascular death [4–6]. The AASI is considered an indirect measure of arterial stiffness. It strongly correlates with both pulse wave velocity and the arterial augmentation index and appears to be a complimentary cardiovascular risk marker to conventional blood pressure indices [7, 8].

Lower nocturnal blood pressure dipping is thought to reflect increased arterial stiffness. Less compliant and ‘stiffer arteries’ are less distensible leading to lower relative nocturnal blood pressure and pulse pressure drop [9]. It would be expected that as a surrogate of arterial stiffness AASI would be linked to blood pressure dipping status, vascular inflammation and adverse cardiovascular events. However, there have been few studies that have examined the relationship between AASI and blood pressure dipping [10–12] and none to investigate its links to inflammation. Whilst increasing AASI has been linked to adverse cardiovascular outcomes and worsening renal function these links and its relative benefits over traditional ABPM measures remain controversial [8, 13, 14].

Vascular inflammation is an established risk marker and key pathogenic mediator in the development of endothelial dysfunction, atherosclerosis and subsequently increasing arterial stiffness [15]. Stimulated white blood cells (WBC) can adhere and penetrate the vascular endothelial intima leading to capillary leukostasis, vascular damage and an increase in arterial stiffness [16, 17]. Several differential WBC ratios have evolved as useful markers of chronic vascular inflammation and been linked to measures of arterial stiffness and major adverse cardiovascular events (MACE) [18–20]. Among the differential white cell indices the greatest evidence exists for the neutrophil–lymphocyte (NLR), platelet-lymphocyte (PLR) and monocyte-lymphocyte ratios (MLR). These measurements have the advantage over many other established inflammatory markers owing to their high reproducibility, low cost determination, widespread availability and ease of measurement. The relationship of AASI and inflammation, to blood pressure dipping status and cardiovascular outcomes have not been examined.

This study had two discrete aims. The first was to investigate the relationship between AASI, vascular inflammation (using lymphocyte ratios) and nocturnal blood pressure dipping status. The second was to examine their association with future MACE. We hypothesised that AASI would be significantly associated with blood lymphocyte ratio counts, blood pressuring dipping status and MACE.

## Methods

### Study population

This study followed the STROBE Initiative Guidelines for cohort studies [21]. This was a single centre retrospective observational study. Consecutive adults aged 18–80 years who underwent 24-h ABPM for the diagnosis of hypertension, or its control, at our institution were included.

Key exclusion criteria were patients with previous organ transplantation, persistent/ permanent atrial fibrillation, a calculated creatinine clearance of < 30 ml/min or known stage IV or V chronic kidney disease (CKD), pregnancy or with active cancer. Patients with severe aortic stenosis, aortic coarctation, active infection or vasculitis, on high dose steroids or who had been hospitalised within the previous week were also excluded.

## 24 Hour ambulatory blood pressure measurement

All tests were performed using an automatic ABPM device (Spacelab 90207, Spacelab Healthcare, Hertford, UK). An automated oscillometric cuff was placed on the non-dominant arm. Blood pressure measurements were set to minute intervals throughout a 24-h recording period. The night-time period was defined as the hours of 22:01 to 06:00 h and the day-time period as 06:01 to 22:00 h. Patients were only included if they had a minimum of 10 day-time and 5 night-time ambulatory blood pressure measures during the 24 h recording period [6, 22]. Patients were advised not to have their ABPM during periods of night shift work.

Nocturnal dipping status was classified into three groups based on the percentage change in mean night time to day time blood pressure as previously defined (a) normal dippers (≥ 10%); (b) non‐dipper (≥ 0% but < 10%) and (c) reverse dipper (< 0%) [6, 23].The AASI was calculated, as previously defined, as 1 minus the regression slope of the diastolic to systolic blood pressure over the 24 h recording period [24]. Increasing AASI values indicate stiffer arteries with all values ranging between 0 and 1.0.

The Primary Endpoint was a composite MACE of cardiovascular death, non-fatal acute limb ischaemia, stroke or transient ischaemic attack (TIA) or acute coronary syndrome (ACS) [25]. Our secondary outcome of interest was cardiovascular death or non-fatal stroke. ACS was defined in accordance with the European Society of Cardiology Guidelines and required diagnostic confirmation by a cardiologist. The diagnosis of stroke or TIA were based on clinical presentation, supported by radiological imaging and had to be confirmed by a stroke physician. Acute limb ischaemia was defined as a sudden decrease in limb perfusion causing a potential threat to limb viability and requiring hospitalisation. Coronary artery disease (CAD) was defined as a previous myocardial infarction, percutaneous coronary angioplasty/stenting, or a significant stenosis of ≥ 70% in ≥ 1 major coronary arteries [26].

### Blood tests

Venous blood for the measurement of lipid profile, full blood count, renal function and Glycated haemoglobin (HbA1c) were analysed in our hospital laboratory and examined within three months of ABPM. The creatinine clearance was calculated using the Cockcroft-Gault Equation [27].

### Ethical approval

This study and its experimental protocol were approved by the Poole Hospital Clinical Research and Innovation Department and the West of Scotland Research Ethics Committee (REC reference: 20/WS/0097). As this was a registry study the need for written informed consent was deemed not to be necessary by the ethics committee.

### Statistical analysis

Statistical analyses were performed with the SPSS 26.0 (SPSS, Chicago, IL, USA) and GraphPad Prism version 6.07 for Windows (GraphPad Software, San Diego, CA, USA). Data inspection and the D'Agostino–Pearson normality were used to assess normality of all continuous data. Continuous data were presented as mean ± standard deviations except for highly skewed data where the median [interquartile range] were shown. Comparisons of continuous data among two groups were performed using an unpaired t test and Mann–Whitney test for normal and non-normally distributed data respectively. Three group comparisons of continuous data (normal, non- and reverse-systolic blood pressure dippers) were performed using a One-way ANOVA and Kruskal–Wallis tests with post-tests for normally and non-normally distributed data respectively. Categorical data were examined using the Fisher’s exact tests and chi squared tests as appropriate. Correlations were investigated using Pearson and Spearman rank coefficients (± 95% confidence interval [CI]) as appropriate. Only notable correlations with a coefficient r ≥ 0.20 were reported.

Associations between prognostic variables and MACE were examined by univariate and multivariate Cox proportional hazards analysis with the results reported as the odds ratio (*B*) and 95% confidence intervals (CI), respectively. Only variables with a *p* value < 0.05 on univariate testing or that were considered clinically relevant with outcomes were entered into the multivariate analyses. To avoid over-fitting a forward LR (likelihood ratio) method were performed with retention set at a significance level of 0.10. Sensitivity analyses were performed to assess the robustness of the final model. A two-sided p value of < 0.05 was considered significant for all comparisons.

### Sample size calculations

This was performed using a proprietary sample-size calculator (GraphPad StatMate version 2.00 for Windows). Published data has suggested an average standard deviation for the AASI of 0.06 to 0.22 among adults with and without hypertension or cardiovascular disease [8]. Based on this data we estimated that a sample size of at least 150 patients per group with and without systolic blood pressure dipping would have > 80% power to detect a difference between mean AASI values of ≥ 0.05 with a significance level (alpha) of 0.05 (two-tailed). This group sample size among non-normal versus normal dippers would have ≥ 80% power to detect a MACE rate of at least 6%.

## Results

### Baseline demographics

A total of 508 patients were included in this study of which 260 (51.2%) were women. The mean age was 58.8 ± 14.0 (range 18–80) years. Overall 98.6% of the population were Caucasian; 66.34% had a previous history of hypertension, 16.9% diabetes mellitus and 8.7% had suffered a previous stroke or TIA. The men (58.4 ± 13.95) and women (59.2 ± 14.1) were of similar age (*p* = 0.55).

### Dipping status and cardiovascular risk factors

From the total cohort 237 (46.7%) patients were classed as normal-dippers, 214 (42.1%) non-dippers and 57 (11.2%) reverse-dippers (Table 1). The reverse-dippers and were older and more likely to have a history of heart failure, peripheral vascular disease (PVD) and diabetes mellitus compared with normal and non-dippers. Creatinine clearance and high-density lipoprotein (HDL) concentrations were significantly lower and NLR and HBA1c levels higher amongst the reverse dippers versus the normal and non-dippers (Table 1). Conversely, the NLR, MLR and HBA1c levels were progressively higher among the non-dippers and reverse-dippers respectively (Table 1). MACE events were more common among the reverse-dippers.

**Table 1:** Relationship between baseline demographics, cardiovascular risk factors and blood marker with blood pressure dipping status

Characteristic	Normal-dippers	Non-dippers	Reverse-dippers	*p* value
Number (%	237 (46.7%)	214 (42.1%)	57 (11.2%)	
Age, years	56.3 ± 13.9	60.5 ± 13.3	62.6 ± 15.4	0.0006^ab^
Male sex (%)	120 (50.6%)	99 (46.3%)	29 (50.9%)	0.61
Height, cm	168.7 ± 10.9	168.5 ± 9.7	168.6 ± 11.7	0.98
Weight, kg	81.9 ± 18.0	82.4 ± 19.6	86.6 ± 24.0	0.27
Body mass index, kg/m^2^	28.8 ± 5.5	29.0 ± 6.1	30.3 ± 6.5	0.26
Coronary artery disease	43 (18.1%)	44 (20.6%)	15 (26.3%)	0.37
Heart failure	6 (2.5%)	9 (4.2%)	8 (14.0%)	0.0008
Peripheral vascular disease	9 (3.8%)	5 (2.3%)	11 (19.3%)	< 0.0001
Diabetes mellitus	24 (10.1%)	43 (20.1%)	19 (33.3%)	< 0.0001
Previous stroke /TIA	21 (8.8%)	11 (5.1%)	8 (14.0%)	0.06
Known hypertension	155 (65.4%)	143 (66.8%)	44 (77.2%)	0.23
Current/ex-smokers	98 (41.4%)	96 (44.9%)	29 (50.9%)	0.40
Ejection fraction, %	59.8 ± 8.2	59.2 ± 9.6	57.5 ± 9.0	0.27
Haemoglobin g/l	141.3 ± 14.9	138.4 ± 15.0	136.5 ± 14.9	0.051
Neutrophil/lymphocyte ratio	2.1 [1.6–2.9]	2.3 [0.7–3.4]	2.6 [2.0–3.9]	< 0.0001^ab^
Platelet/lymphocyte ratio	145.3 ± 75.8	154.7 ± 70.5	167.2 ± 92.2	0.11
Monocyte/lymphocyte ratio	0.32 ± 0.16	0.33 ± 0.17	0.37 ± 0.18	0.13
Creatinine clearance	76.8 ± 26.7	72.1 ± 28.1	70.5 ± 37.3	0.016^b^
Total cholesterol, mmol/l	4.9 ± 1.1	4.7 ± 1.2	4.6 ± 1.0	0.18
HDL cholesterol, mmol/l	1.56 ± 0.5	1.49 ± 0.5	1.38 ± 0.4	0.037^b^
Triglycerides, mmol/l	1.52 ± 0.9	1.57 ± 0.9	1.73 ± 1.0	0.17
HBA1c, mmol/mol	41.2 ± 12.1	43.9 ± 12.6	49.39 ± 21.3	0.0008^bc^
MACE events	15 (6.3%)	15 (7.0%)	9 (15.8%)	0.049

TIA, transient ischaemic attack; HBA1c Glycosylated haemoglobin; HDL, high density lipoprotein

*p* values refer to the results of overall comparison between the three groups: 1. Normal dippers, 2. Non-dippers and 3. Reverse-dippers. Significant difference on post hoc tests a. normal vs. non dippers, b. normal vs. reverse dippers, c. non vs. reverse dippers

**Fig. 1:**
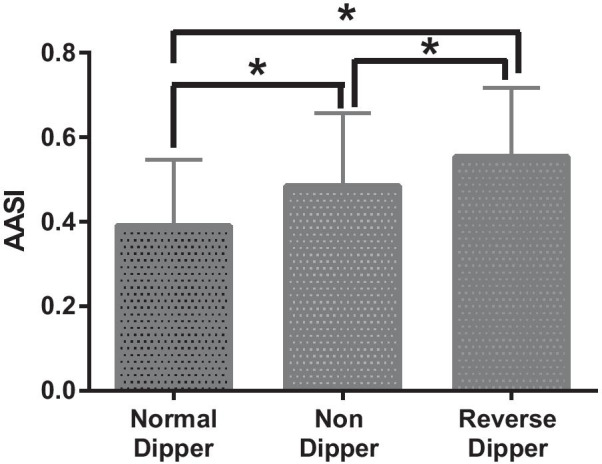
Relationship between AASI and systolic blood pressure dipping status (*refers to significance between groups)

Non-dippers and reverse-dippers had significantly higher 24 h average systolic blood pressure and pulse pressure than normal-dippers. Non and reverse-dippers had higher AASI (Fig. 1), night-time systolic, diastolic, mean arterial blood pressure and pulse pressures compared with the normal dippers respectively (Table 2). Day-time diastolic blood pressure and mean arterial blood pressures were higher yet pulse pressure lower in normal-dippers versus non- and reverse-dippers respectively.

**Table 2:** Relationship of ambulatory blood pressure parameters to systolic blood pressure dipping status

Characteristic	Normal dippers	Non dippers	Reverse dippers	*p* value
*24 h ABPM averages*				
Systolic blood pressure, mmHg	129.6 ± 14.1	133.9 ± 15.6	135.4 ± 15.9	0.002^ab^
Diastolic blood pressure, mmHg	77.0 ± 9.7	76.1 ± 10.6	74.3 ± 11.7	0.18
Mean arterial blood pressure, mmHg	95.1 ± 9.8	96.3 ± 10.5	95.8 ± 11.8	0.49
Pulse pressure, mmHg	52.6 ± 11.8	57.8 ± 13.3	61.2 ± 10.9	< 0.0001^ab^
*Day-time averages*				
Systolic blood pressure, mmHg	135.4 ± 14.9	135.9 ± 15.7	134.0 ± 15.9	0.71
Diastolic blood pressure, mmHg	81.3 ± 10.3	77.9 ± 10.6	74.2 ± 11.8	< 0.0001^ab^
Mean arterial pressure, mmHg	99.9 ± 10.4	98.1 ± 10.5	95.3 ± 12.0	0.008^b^
Pulse pressure, mmHg	54.1 ± 12.60	57.9 ± 13.5	59.8 ± 10.7	0.0009^ab^
*Night-time averages*				
Systolic blood pressure, mmHg	114.2 ± 12.0	128.1 ± 15.2	139.9 ± 16.3	< 0.0001^abc^
Diastolic blood pressure, mmHg	65.6 ± 8.6	70.3 ± 9.7	74.7 ± 11.4	< 0.0001^abc^
Mean arterial pressure, mmHg	82.7 ± 8.8	90.9 ± 10.3	97.6 ± 11.6	< 0.0001^abc^
Pulse pressure, mmHg	48.7 ± 10.7	57.8 ± 12.8	65.2 ± 12.7	< 0.0001^abc^
Systolic blood pressure dip, %	14.2 [12.2–18.4]	6.0 [3.2 to 8.3]	− 3.8 [− 6.6 to − .2]	< 0.0001^abc^
*AASI*	0.39 ± 0.16	0.48 ± 0.17	0.56 ± 0.16	< 0.0001^abc^

ABPM Ambulatory blood pressure monitoring; AASI, Ambulatory arterial stiffness index

*p* values refer to the results of overall comparison between the three groups: 1. normal dippers, 2. non-dippers and 3. reverse dippers. Significant difference on post hoc tests: a. Normal vs. non-dippers, b. Normal vs. reverse-dippers, c. Non vs. reverse-dippers

### Relationship between AASI to inflammation, dipping status and other variables

AASI positively correlated with age (r = 0.48; 0.41 to 0.54; *p* < 0.0001), HBA1c (r = 0.30; 0.21 to 0.39: *p* < 0.0001), NLR (r = 0.20; 0.11 to 0.29: < 0.0001) and systolic blood pressure (r = 0.26; 0.17 to 0.34: *p* < 0.0001). AASI inversely correlated with creatinine clearance (r = − 0.32; − 0.40 to − 0.23: *p* < 0.0001), diastolic blood pressure (r = − 0.23; − 0.32 to − 0.15: *p* < 0.0001) systolic blood pressure dipping % (r = − 0.34; − 0.42 to − 0.26: *p* < 0.0001), diastolic blood pressure dipping % (r = − 0.48; − 0.55 to − 0.41: *p* < 0.0001) and MAP blood pressure dipping % (r = − 0.44; − 0.51 to − 0.36: *p* < 0.0001).

The AASI was significantly greater in women versus men (0.47 ± 0.17 vs. 0.43 ± 0.17; *p* = 0.02). AASI values were also higher in those with, versus without respectively, a history of CAD (0.49 ± 0.16 vs. 0.44 ± 0.18; *p* = 0.003), diabetes mellitus (0.55 ± 0.15 vs. 0.43 ± 0.17; *p* < 0.0001), previous stroke/TIA (0.54 ± 0.19 vs. 0.44 ± 0.19; *p* = 0.0008), PVD (0.52 ± 0.14 vs. 0.44 ± 0.17; *p* = 0.034), known hypertension (0.46 ± 0.17 vs. 0.42 ± 0.17; *p* = 0.004).

### Outcome data

Overall 39 (7.7%) patients had one or more adverse cardiovascular events which included a total of seven cardiovascular deaths and 14 non-fatal strokes/TIAs. The mean follow up (to death or analysis) was 113.7 ± 64.0 weeks.

The univariate predictors of MACE were age, history of stroke/TIA, heart failure, coronary disease, hypertension and PVD, AASI, lower systolic blood pressure dipping (%), mean arterial blood pressure dipping (%) and 24 h pulse pressure, NLR, MLR, creatinine clearance, and lower haemoglobin (Table 3).

**Table 3:** Results of univariate and multivariate Cox regression analysis for MACE

Characteristic	Univariate analysis		Multivariate analysis	
	Odds ratio (95% CI)	*p* value	Odds ratio (95% CI)	*p* value
Age, years	1.07 (1.03–1.10)	< 0.0001	1.04 (1.001–1.08)	0.044
Male sex (%)	0.73 (0.38–1.37)	0.33	–	NS
Coronary artery disease	2.333 (1.20–4.50)	0.013	–	NS
Heart failure	5.62 (2.47–12.81)	< 0.0001	5.65 (2.36–13.55)	< 0.0001
Diabetes mellitus	2.50 (1.25–4.90)	0.010	–	NS
Previous stroke /TIA	3.20 (1.40–7.30)	0.006	2.49 (1.05–5.93)	0.039
Known hypertension	3.26 (1.27–8.34)	0.014	–	NS
Peripheral vascular disease	4.61 (2.02–10.51)	< 0.0001	3.0 (1.27–7.10)	0.013
Haemoglobin	0.97 (0.95–0.98)	0.002	0.98 (0.96–0.99)	0.045
Neutrophil/lymphocyte ratio	1.17 (1.08–1.28)	< 0.0001	1.13 (1.02–1.24	0.02
Monocyte/lymphocyte ratio	1.02 (1.01–1.04)	< 0.0001	–	NS
Creatinine clearance	0.98 (0.97–0.99)	0.01	–	NS
Systolic blood pressure dip, %	0.95 (0.91–0.98)	0.012	–	NS
AASI	1.03 (1.01–1.05)	0.006	–	NS

TIA, transient ischaemic attack; HBA1c Glycosylated haemoglobin; AASI, ambulatory arterial stiffness index; NS, non-significant

The only independent predictors of MACE were increasing age, a history of heart failure, PVD, previous stroke/TIA, lower haemoglobin and increasing NLR (overall model Chi-square 60.67; *p* < 0.0001) (Table 3). Previous stroke/TIA, MLR (OR 1.04; 1.02–1.06: *p* = 0.009), PVD and female sex were independent predictors of future cardiovascular death or non-fatal stroke/TIA (overall model Chi-square 37.08; *p* < 0.0001). The full database is available in the supplement (Additional file 1).

## Discussion

This was first study to investigate the relationship between AASI, vascular inflammation and blood pressure dipping and their association to adverse cardiovascular outcomes. We found that a reduced blood pressure dipping pattern was linked to higher AASI and measures of vascular inflammation (MLR and NLR). Systolic blood pressure dipping %, AASI, MLR and NLR were associated with MACE on univariate analyses. Increased NLRs were independently associated with MACE and MLR with cardiovascular death or non-fatal stroke/TIA.

To our knowledge this is the largest and one of the few studies to investigate the relationship between systolic blood pressure dipping and vascular inflammation using lymphocyte ratios. We observed an inverse relationship between the neutrophil and monocyte-lymphocyte ratios and systolic blood pressure dipping. Higher ratios were associated with non- and reverse blood pressure dipping, which are recognised risk markers of increased cardiovascular risk [5]. Previously, Sabul et al. (n = 166) observed higher NLR in patients among non-dipper versus with dipper adult hypertensives respectively. In another study, Ahbap et al. observed higher NLR among dipper versus non dipper hypertensives with CKD supporting our results. The MLR is rapidly emerging as another important marker of vascular inflammation has been linked to carotid stenosis severity in ischaemic stroke and to the severity of coronary disease and cardiovascular mortality [28–30]. There was no association between PLR and either blood pressure dipping status or MACE in our cohort.

Chronic vascular inflammation and the infiltration of the vascular endothelium with leukocyte subsets is one of the primary driving forces in the development of atherosclerotic cardiovascular disease [31]. Lipid accumulation within the vascular wall intima leads to passage of stimulated monocytes, which are major precursors of macrophage, passage across the endothelium [31]. Neutrophils and platelets also influence atherosclerosis and its complications by intensifying inflammation with platelets also contributing to clot formation. We found that the NLR was an independent predictor of our primary MACE and the MLR independently predicted cardiovascular mortality as well as the composite of cardiovascular mortality and non-fatal stroke or TIA. These results add further weight to the current evidence supporting the potential utility of lymphocyte biomarkers in cardiovascular risk assessment. They have the advantage over many traditional markers due to their low cost, widespread use and the rapid availability of results.

Our interest in the AASI stems from its easy computational availability with automated results included in our standardised reports, coupled most importantly with its cited role as a potential indirect marker of arterial stiffness. All of the factors that we found to be associated with AASI and its higher levels in this study are recognised risk factors for increased arterial stiffness, with one exception. We observed that women had significantly higher AASI values than men despite their similar ages. This could relate, in part, to differences in their comorbidities and relative burden of cardiovascular risk factors (eg hypertension, lipid profiles etc.). A detailed comparison was beyond the scope of this study. It is nevertheless notable that it has been reported that women exhibit greater age related increase in measures of large artery stiffness than men, supporting our findings [32–34].

The relationship between AASI and systolic blood pressure dipping status has only been previous examined in a few studies with conflicting results [10, 12]. We found that AASI values were progressively and significantly higher in non-dippers and reverse dippers versus normal dippers. Moreover, dipping status was an independent predictor of AASI and likely increased arterial stiffness.

This study has a number of limitations that should be acknowledged. This was a retrospective single centre study and hence prone to potential bias. Only outcomes registered with the local hospitals could be monitored and hence we cannot exclude the possibility that some patients could have presented to another hospital with a cardiovascular event outside of our locality. However, these numbers are likely to be very small given that only local and registered patients were included and there were on-going active records available for the vast majority of patients. All 24-h ABPM were set to similar times for the determination or day-time and night-time blood pressures and its associated indices. Given the wide range of ages of patients included in this study it is likely that there would have been periods graded as effectively night-time when the patients were still very much awake and day-time when patients may have been asleep. The use of a wide fixed night-time period in this study could have led to inclusion of transition periods in the morning and evening when blood pressure changes rapidly leading to the potential misclassification of dipping status [35]. However, at present there is a lack of consensus on the optimal nocturnal recording period to determine dipping status [2, 3, 23]. Furthermore, the links between non/reverse dipping status and MACE have been observed with various classifications including wide fixed night-time periods as in this study [6, 36]. Finally whilst our sample size was reasonable our event rates were relatively low and hence, despite conducting sensitivity testing, we cannot exclude the potential for bias.

In conclusion, in this study we found that that AASI was significantly correlated with NLR and inversely related to systolic blood pressure dipping. Systolic blood pressure dipping, AASI, the neutrophil and monocyte-lymphocyte ratios were univariate predictors of MACE. The NLR was an independent predictor of the primary MACE, and MLR for cardiovascular death or non-fatal stroke. This data endorses the association between AASI, inflammation and cardiovascular risk.

## Supplementary Information

**Additional file 1.** The full database supplement

## Data Availability

All data generated or analysed during this study are included in this published article and its supplementary information files.
